# Investigating the Effect of Recruitment Variability on Length-Based Recruitment Indices for Antarctic Krill Using an Individual-Based Population Dynamics Model

**DOI:** 10.1371/journal.pone.0114378

**Published:** 2014-12-03

**Authors:** Stéphane Thanassekos, Martin J. Cox, Keith Reid

**Affiliations:** 1 Commission for the Conservation of Antarctic Marine Living Resources Secretariat, Hobart, Tasmania, Australia; 2 Southern Ocean Ecosystem Change, Australian Antarctic Division, Kingston, Tasmania, Australia; Institute of Marine Research, Norway

## Abstract

Antarctic krill (*Euphausia superba*; herein krill) is monitored as part of an on-going fisheries observer program that collects length-frequency data. A krill feedback management programme is currently being developed, and as part of this development, the utility of data-derived indices describing population level processes is being assessed. To date, however, little work has been carried out on the selection of optimum recruitment indices and it has not been possible to assess the performance of length-based recruitment indices across a range of recruitment variability. Neither has there been an assessment of uncertainty in the relationship between an index and the actual level of recruitment. Thus, until now, it has not been possible to take into account recruitment index uncertainty in krill stock management or when investigating relationships between recruitment and environmental drivers. Using length-frequency samples from a simulated population – where recruitment is known – the performance of six potential length-based recruitment indices is assessed, by exploring the index-to-recruitment relationship under increasing levels of recruitment variability (from ±10% to ±100% around a mean annual recruitment). The annual minimum of the proportion of individuals smaller than 40 mm (F40 min, %) was selected because it had the most robust index-to-recruitment relationship across differing levels of recruitment variability. The relationship was curvilinear and best described by a power law. Model uncertainty was described using the 95% prediction intervals, which were used to calculate coverage probabilities and assess model performance. Despite being the optimum recruitment index, the performance of F40 min degraded under high (>50%) recruitment variability. Due to the persistence of cohorts in the population over several years, the inclusion of F40 min values from preceding years in the relationship used to estimate recruitment in a given year improved its accuracy (mean bias reduction of 8.3% when including three F40 min values under a recruitment variability of 60%).

## Introduction

Krill is an important link between lower trophic levels (phytoplankton) and high-order predators such as penguins and whales, in the Antarctic marine ecosystem [1]. Krill has also been the focus of both long-term scientific research and commercial fishery (e.g. [2]–[4]). Multiple sources of data from scientific surveys and the fishery have generated databases that provide information on key life-history characteristics of krill such as growth, mortality and recruitment. Typically, scientific research on krill has focussed on the summer period when logistics and operational factors are more amenable; however, the commercial fishery for krill operates year-round [5]. The Commission for the Conservation of Antarctic Marine Living Resources Scheme of International Scientific Observation (CCAMLR SISO; www.ccamlr.org), was initiated in 1992 to collect data from the krill fishery, including representative length-frequency data, from commercial captures on board fishing vessels. Recent increases in observer coverage levels in the krill fishery [6] has provided an increase in the data available from the fishery both spatially and temporally. The database of krill lengths represents an opportunity to investigate krill population dynamics, at scales not typically feasible using data from scientific surveys.

Depending on the areas, the longevity of krill in the wild is estimated to range between 4 and 7 years, with an age at maturity of about 3 years [7]. Given the relatively short life-cycle and relatively high mortality rate of krill, variation in the level of recruitment is a major contributor to inter-annual variability in the abundance of krill (e.g. [8]). Measuring recruitment directly (where all new individuals in the population are recorded) in wild populations is typically only possible in a very small number of closed terrestrial systems (e.g. St Kilda Soay sheep [9]) and is impractical for marine taxa. Several studies of krill have developed methods to estimate recruitment based on changes in the population size-structure using length measurements of individual krill caught with nets (e.g. [10], [11]). The rationale behind these methods is that recruitment (i.e. the number of one-year old individuals entering the population) can be estimated based on the increase in the proportion of smaller (and by inference younger) individuals in the population. These proportional indices of recruitment have been instrumental in investigating krill population dynamics (e.g. [12]–[14]) and ecosystem processes (e.g. [15]–[17]), and more recently, within integrated assessment frameworks [18], [19]. Quantifying the relationship between inter-annual changes in recruitment and in environmental variables such as ice-cover [20]–[22] and ocean currents [13], [23], is crucial to our understanding of the drivers of population dynamics, and enables extrapolation to future krill population states. Such analyses, however, rely on assumptions about the relationship between absolute population recruitment and proportional indices of recruitment derived from length-frequency data.

In many studies, krill recruitment is estimated using a proportional index ‘R1’ defined as the ratio of the number of 1-year-old individuals to the total number of individuals (e.g. [12], [24]). This ratio can be calculated by using maximum likelihood to fit age-specific mixtures of normal distributions to population level length distribution data [10]. As there are currently no cost-effective and precise methods to age krill [25], the allocation of modes in length distributions to age-classes is dependent upon an underlying growth model. Therefore while there is no practical method to estimate absolute recruitment using length frequency data, there is a need develop and validate alternative methods for that purpose.

Ideally, for a population with constant recruitment, a length-based index should accurately reflect recruitment and changes in recruitment should be reflected in changes in the index. That said, the relationship between krill recruitment indices and absolute recruitment has to date not been quantified and this has two important implications. Firstly, the performance of a given index – that is how accurately an index represents absolute recruitment – is unknown. In an extreme example, this may lead to an index returning the same estimated recruitment under low or high recruitment. In this circumstance, the recruitment index would contain no, or misleading information. Secondly, krill recruitment varies inter-annually (e.g. [2]) and it is extremely unlikely that a given recruitment index will perform equally well across all levels of biologically plausible ranges of recruitment variability. Indeed, a priori it is reasonable to expect the performance of recruitment indices to decrease with increasing recruitment variability especially where the absolute level of recruitment is not affected by recruitment in the previous year whereas a relative index is. Nevertheless, as it is not possible to determine recruitment variability directly, it is important that a recruitment index performs adequately across the largest range of recruitment variability.

Since recruitment and its variability cannot be observed directly, regression analyses based on simulated data offer a means to examine the relationship between a length-based index and recruitment and especially to investigate the uncertainty arising from increased recruitment variability.

The relationships between recruitment and length-based indices of recruitment of krill were investigated at differing levels of recruitment variability in a population simulated using an individual-based model. In order to produce results that have direct relevance to the interpretation of the data collected as part of the CCAMLR SISO, individual krill were subsampled within the model according to a length-dependent selectivity function estimated for krill commercial fishing gear [26].

The specific goals of this work were to: (i) investigate the relationships between length-based indices and recruitment, (ii) select an optimum recruitment index from a suite of recruitment indices under various levels of recruitment variability; (iii) use a regression analysis to determine the relationship between the recruitment index and absolute recruitment; (iv) determine the performance of the selected recruitment index, and (v) reduce uncertainty in the recruitment index-absolute recruitment relationship by including consecutive index values from preceding years.

## Methods

### 2.1 Candidate length-based recruitment indices

**Figure 1:**
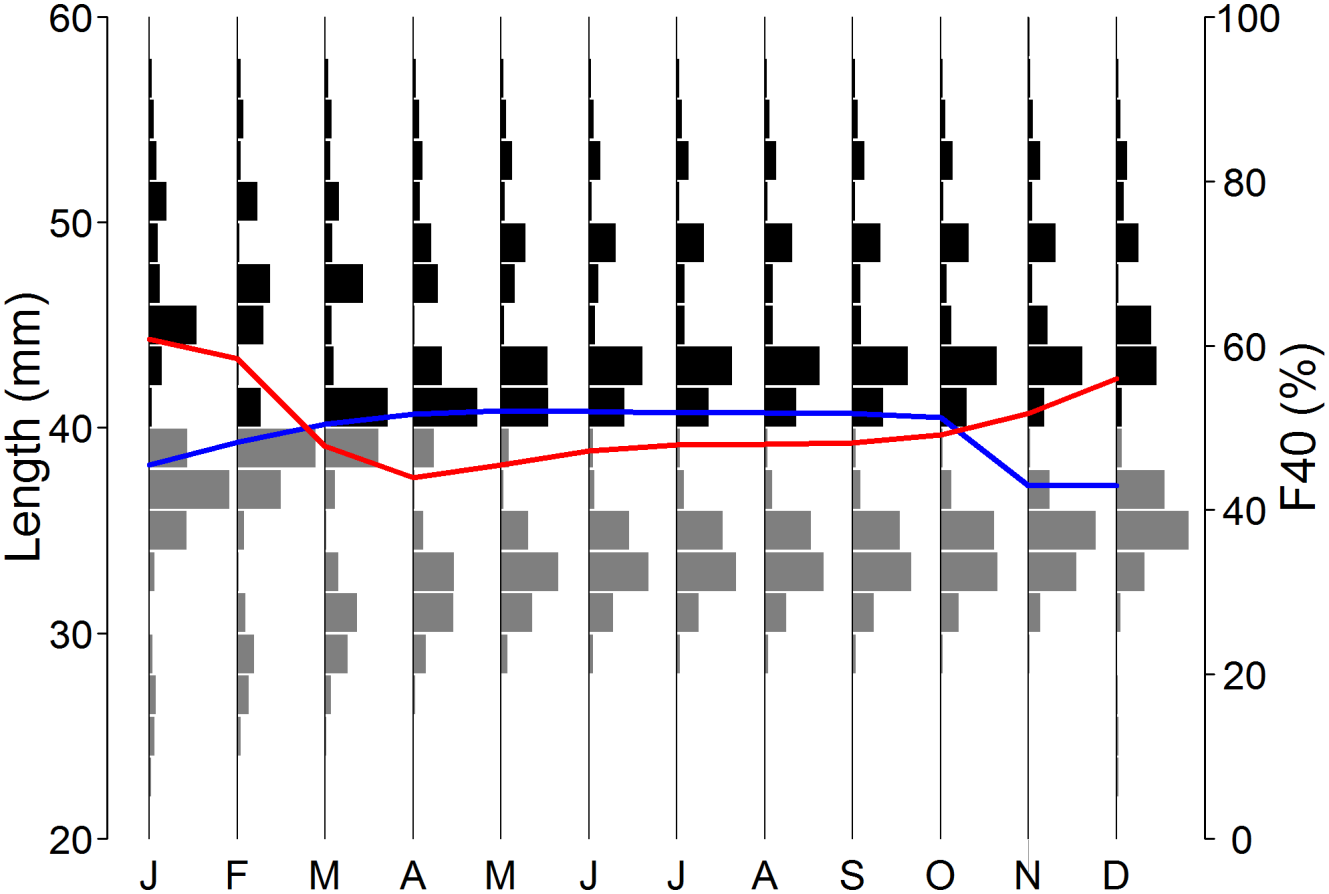
*Euphausia superba*. Deriving recruitment indices from length frequency distributions. Monthly length-frequency distributions simulated in the last year of ten-year population dynamics with constant summer recruitment of 4×10^6^ individuals. Grey histograms indicate the frequencies of individuals smaller than 40 mm, the blue line indicates the monthly median length (mm) and the red line indicates the proportion of individuals smaller than 40 mm (F40, %).

Based on length frequency distributions, recruitment can be estimated using order statistics. Two order statistics, the median length (mm) and the proportion of individuals smaller than 40 mm (F40, %) were used in this investigation (Fig. 1). The size of 40 mm was chosen as an appropriate cut-off to segregate recruits from older cohorts, once recruits became dominant in length frequency distributions (Fig. 1, after April). Using a cut-off size instead of fitting a normal distribution to each length frequency mode [10] was chosen as a simpler and less ambiguous approach when compared to the often difficult and sometimes subjective task of determining modes using observations. Recruitment (i.e. the sum of one-year old individuals entering the population in a given year) is a single annual event while length-frequencies – and therefore length-based indices – are known to vary at the sub-annual scale (e.g. [3], [11]). Typically krill recruitment is summarised as an annual index [10]–[12], therefore, the monthly recruitment indices, median length (mm) and F40 (%) are summarised by calculating their annual minimum, annual maximum and annual span (maximum-minimum), resulting in six candidate indices of annual recruitment. Using a krill population dynamics model in which recruitment was set randomly each year, the distribution of each index as a function of recruitment was investigated to determine which index would provide the optimum indicator of recruitment.

### 2.2 Simulations

**Figure 2:**
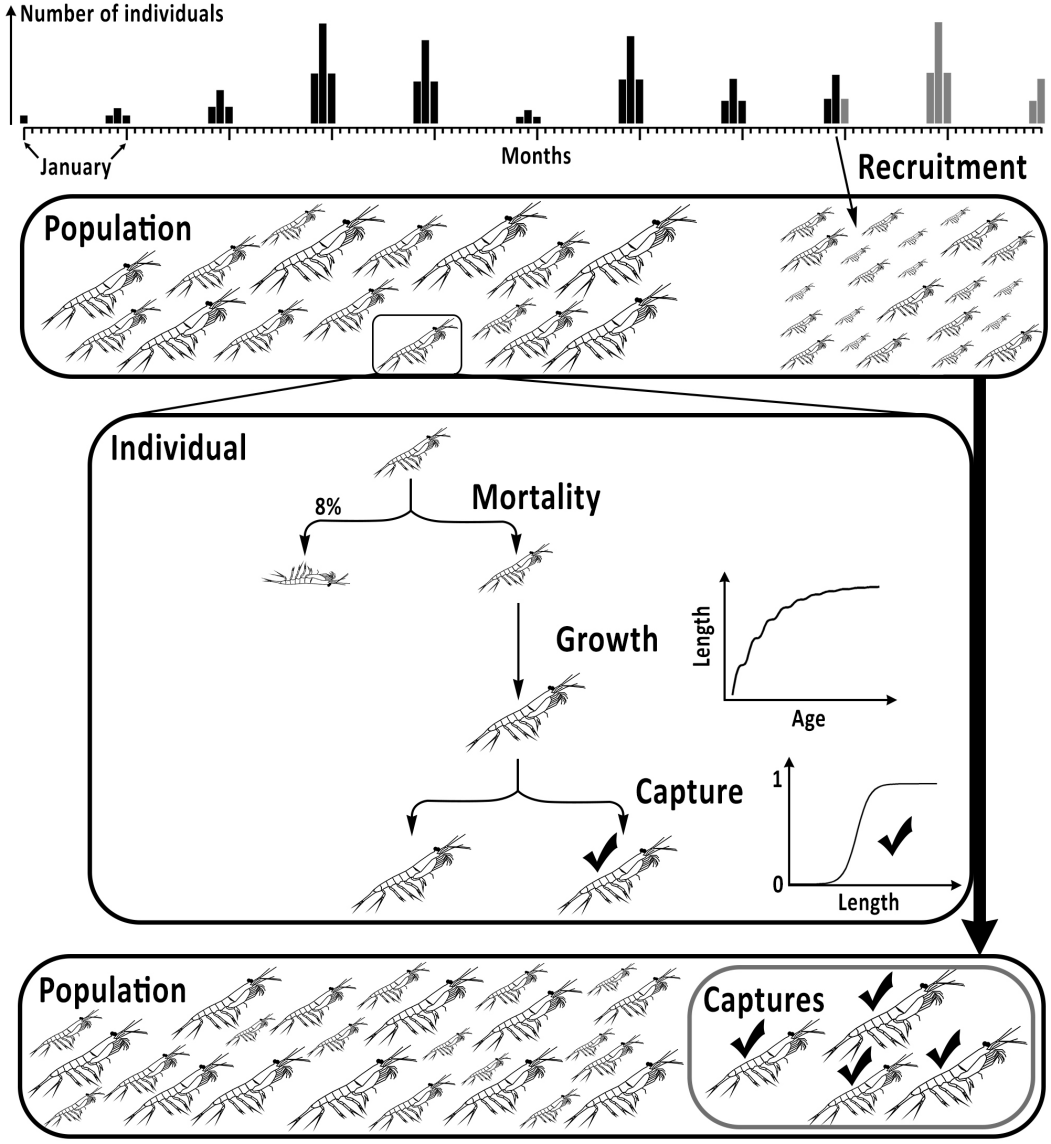
Antarctic krill (*Euphausia superba*) population model flow chart of monthly computations (here December of the 8^th^ year) in a given ten-year simulation. Summer recruitment is simulated by releasing a random number of recruits each year in the population (25% in November and January and 50% in December). For each individual, mortality, growth and capture are computed sequentially (see text for details), and, all individuals available for capture are included in the computation of length-based recruitment indices.

Krill recruitment and its variability cannot be observed directly, so a model of krill population dynamics (Fig. 2), parameterised using values from the primary literature was used to simulate biologically plausible krill populations under various levels of recruitment variability.

#### 2.2.1 Simulating a krill population

The model (Fig. 2), developed using R 3.0.3 [27], had a monthly resolution and for each individual in the population, the likelihood of survival, the growth increment and the probability of capture by the fishery were sequentially computed at each time-step. The population was tracked for ten years and in each ten-year simulation a random number of recruits were released each summer. During each simulation, the monthly median length and F40 were computed from all individuals available for capture by the fishery. The number of recruits entering the population and the monthly median length and F40 values in the final year were calculated and stored. Increasing levels of recruitment variability were achieved by releasing a number of recruits randomly set around a mean 4×10^6^ individuals over a range increasing from ±10% to ±100% by 10% increments (i.e. 10 levels of recruitment variability). For each level of recruitment variability, 2,000 simulations were run, resulting in a total of 20,000 ten-year simulations. The model outputs were used to investigate the link between recruitment and each of the recruitment indices (Section 2.1) under different levels of recruitment variability.

*Recruitment*: Annual recruitment was simulated by releasing a random number of 1-year old individual krill into the model over the course of the summer period (25% in November and January and 50% in December).

*Mortality*: Siegel [7] reviewed krill life history parameters and determined realistic estimates of natural mortality ranged between 0.66 yr^−1^ and 1.35 yr^−1^ (mean = 1.0 yr^−1^). To apply this mean rate in our model it must be converted via the following relationship [28]:
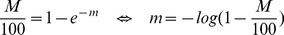
(1)where *M* is the proportional rate used in the individual-based model (in % time^−1^) and *m* is the exponential decay rate used in population dynamics models (in time^−1^); in this case, a mortality rate of 1.0 yr^−1^ or 0.0833 month^−1^ corresponds to 8% month^−1^. A constant mortality rate of 8% month^−1^ was therefore used to determine the transition of each individual between time-steps. At each time-step, a probability *P_M_* was drawn at random from a uniform distribution bound between 0 and 100%, and where *P_M_*>*M* the individual survived and entered the next time-step. Upon entry into the next time-step, the age of the individual was incremented by a month.

*Growth*: Each recruit was assigned an initial length drawn at random from a normal distribution (mean = 21.742 mm, standard deviation = 2 mm); the initial mean estimated using a von Bertalanffy growth curve commonly used for krill [29], and a standard deviation resulting in realistic dispersions of lengths around each mode (Fig. 1). Subsequent individual growth was computed at each time-step using a seasonally-varying von Bertalanffy growth model in line with the model presented by Siegel (1987 [30]; See Information S1).

#### 2.2.2 Simulating capture by the fishery

The proportion of individuals available for capture by the fishery was determined by a length-dependent selectivity function. An individual was considered to be available for capture based on the commercial fishery selectivity ogive given in Krag et al. (2014 [26]) such that at each time-step, a probability *P_S_* was drawn at random from a uniform distribution bound between 0 and 1 and the individual was available when:
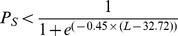
(2)where *L* is the length of the individual. No further sub-sampling (i.e. inclusion of sampling error) was applied; therefore all surviving individuals that were available for capture by the fishery were included in the computation of monthly length-based indices (but not removed from the population). The selectivity ogive used in the model is the best currently available estimate for commercial krill fishing gear. It is however important to note that it is based on a 15.4 mm diamond mesh size [26] and that our findings would only apply for krill sampled with a gear of similar mesh size and type.

#### 2.2.3 Simulating recruitment variability

Different levels of recruitment variability were simulated within bounds defined by a recruitment variability amplitude (Rvar). The number of recruits released each year in the model R_(y)_ was computed as the sum of a mean value R_m_ and a deviation R_d_ (R_(y)_  = R_m_+R_d_). Deviations of different amplitude were achieved using a number drawn at random (R_R_, %) from a uniform distribution bound between –Rvar and +Rvar; with Rvar (%) corresponding to a recruitment variability amplitude ranging from 10% to 100% and:
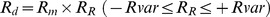
(3)


For example, with R_m_ = 4×10^6^ and Rvar = 50%, the number of recruits released in a given year was randomly set between 2×10^6^ and 6×10^6^ (i.e. 4×10^6^±50%). Using a mean recruitment of R_m_ = 4×10^6^, each Rvar value (10% to 100% by 10% increment) was used in 2000 simulations of ten-year krill population dynamics. The numbers of individuals (4×10^6^) and simulations (2000) enabled producing a sufficiently representative set of simulations and individual histories to investigate the effect of recruitment variability on the population size structure. The ten-year duration of each simulation ensured reaching population stable state under constant recruitment.

### 2.3 Selecting the optimum recruitment index

Within the krill population model, recruitment variability, absolute recruitment and the corresponding values of each length-based index are known. Comparing recruitment to each recruitment index under different levels of recruitment variability, the performance of each index was assessed using two criteria:

- the recruitment index is monotonically related to absolute recruitment – this is important as no other information can be used determine absolute recruitment, so any underlying absolute recruitment to recruitment index relationship must be capable of being predicted using simple (single explanatory variable) regression, and
- the recruitment index is unbiased across all ranges of recruitment variability. This is important because the variability of recruitment in reality is unknown, so the relationship between recruitment and the recruitment index should ideally, remain unchanged under any level of recruitment variability.

### 2.4 Predicting recruitment using a recruitment index

Once the optimum length-based recruitment index, *I*, was found amongst those tested, a simple formula, R = *f(I)*, to estimate recruitment as a function of that index was determined by regression analysis. A regression analysis was performed on the model outputs (recruitment versus index values). For each amplitude of recruitment variability the change in performance of the index as a function of recruitment variability was assessed.

### 2.5 Assessing predictive performance

The purpose of *f(I)* is predictive, and is not intended for inference. In order to assess the predictive performance of *f(I)*, the prediction error (%) was computed using:
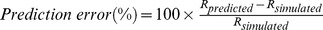
(4)


In addition, the performance of *f(I)* in capturing recruitment uncertainty was assessed at each level of recruitment variability by computing coverage probability, here defined as the percentage of simulated recruitment values that fell inside the 95% predicted recruitment intervals.

### 2.6 Predicting recruitment using past index values

A recruitment event can potentially impact krill population size structure over several years, and additional information describing current recruitment may be contained in index values from previous years. Using the optimum length-based recruitment index from the candidate indices, the relationship between recruitment in the last year of simulations and values of that index in preceding years was investigated. For instance, given a formula (*f*) between recruitment (R) and an index *I* on year 10 (*y_10_*):
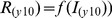
(5)


A multiannual formula where:
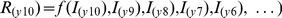
(6)was determined by multiple regressions of the model outputs. The effect of the inclusion of an increasing number of consecutive index values was assessed through changes in the prediction error (Eq. 4) computed for the regressions under three selected amplitudes of recruitment variability (Rvar = 30%, 60%, 90%).

## Results

### 3.1 Selecting a recruitment index

**Figure 3:**
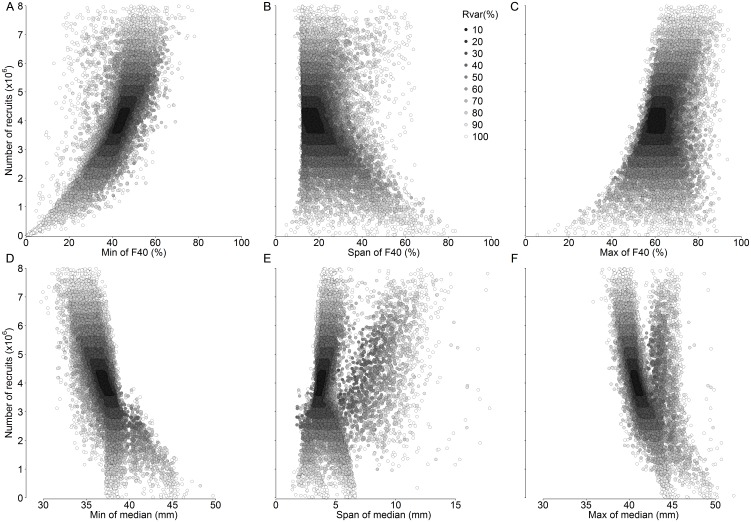
*Euphausia superba*. Simulated recruitment to recruitment index relationship under contrasting levels of recruitment variability (Rvar, %; see section 2.2.3). Model outputs are provided for the last year of 2,000 ten-year simulations per Rvar value.

The selection of the optimum recruitment index from the six candidate indices was based on (i) the distribution of index values as a function of recruitment and (ii) the impact of recruitment variability on these distributions (Fig. 3). The indices derived from F40 – the proportion of individuals smaller than 40 mm – had a monotonic relationship with absolute recruitment across all ranges of recruitment variability (Fig. 3A–C), making F40 indices potentially useful measures of krill recruitment. The indices derived from the median length had more complex relationships with absolute recruitment (Fig. 3D–F). The span and maximum of the median (Fig. 3E, F) had highly non-monotonic responses, and were eliminated as potential indices.

Out of all indices considered, the minimum F40 index (Fig. 3A) followed the clearest monotonic trend with recruitment and provided the strongest differentiation between low and high recruitment. In contrast to this, the span of F40 index (Fig. 3B) covered a wide range of recruitment values, making a regression analysis problematic. The maximum of F40 index (Fig. 3C) had poor coverage of lower recruitment values and had no clear relationship with recruitment. The minimum of median index (Fig. 3D) had difficulty accounting for lower recruitment with high index variability when absolute recruitment was less than 3×10^6^ individuals.

Increasing recruitment variability resulted in an increased variability in all indices. Amongst all indices, the minimum F40 had the lowest variability across all levels of recruitment variability. Furthermore, the relationship between recruitment and minimum F40 was consistently following a curvilinear trend across levels of recruitment variability.

The minimum F40 (*F40 min*) was therefore selected as the optimum recruitment index, and its relationship with recruitment (*R*) was best described using a linear regression of log-transformed values (i.e. a power law), with an intercept (β_0_) and a slope (β_1_):
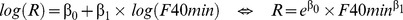
(7)


Subsequent analyses are carried out on the *F40 min* index.

### 3.2 Regression predictive performance

**Figure 4:**
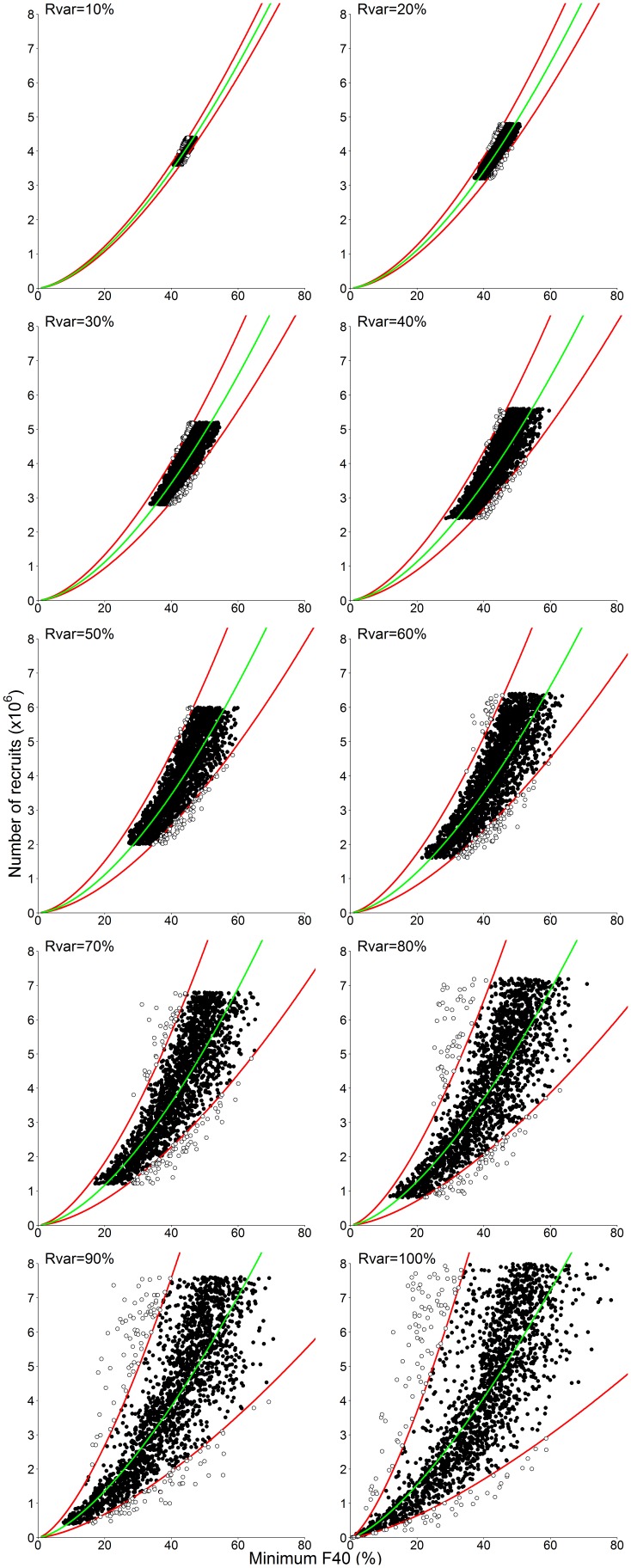
*Euphausia superba*. Curvilinear F40 min regression fits (green line, Eq. 7) for each amplitude of recruitment variability. Recruitment values (y-axes) falling outside of the 95% prediction intervals (red lines) are shown as white points, those inside as black points.

**Figure 5:**
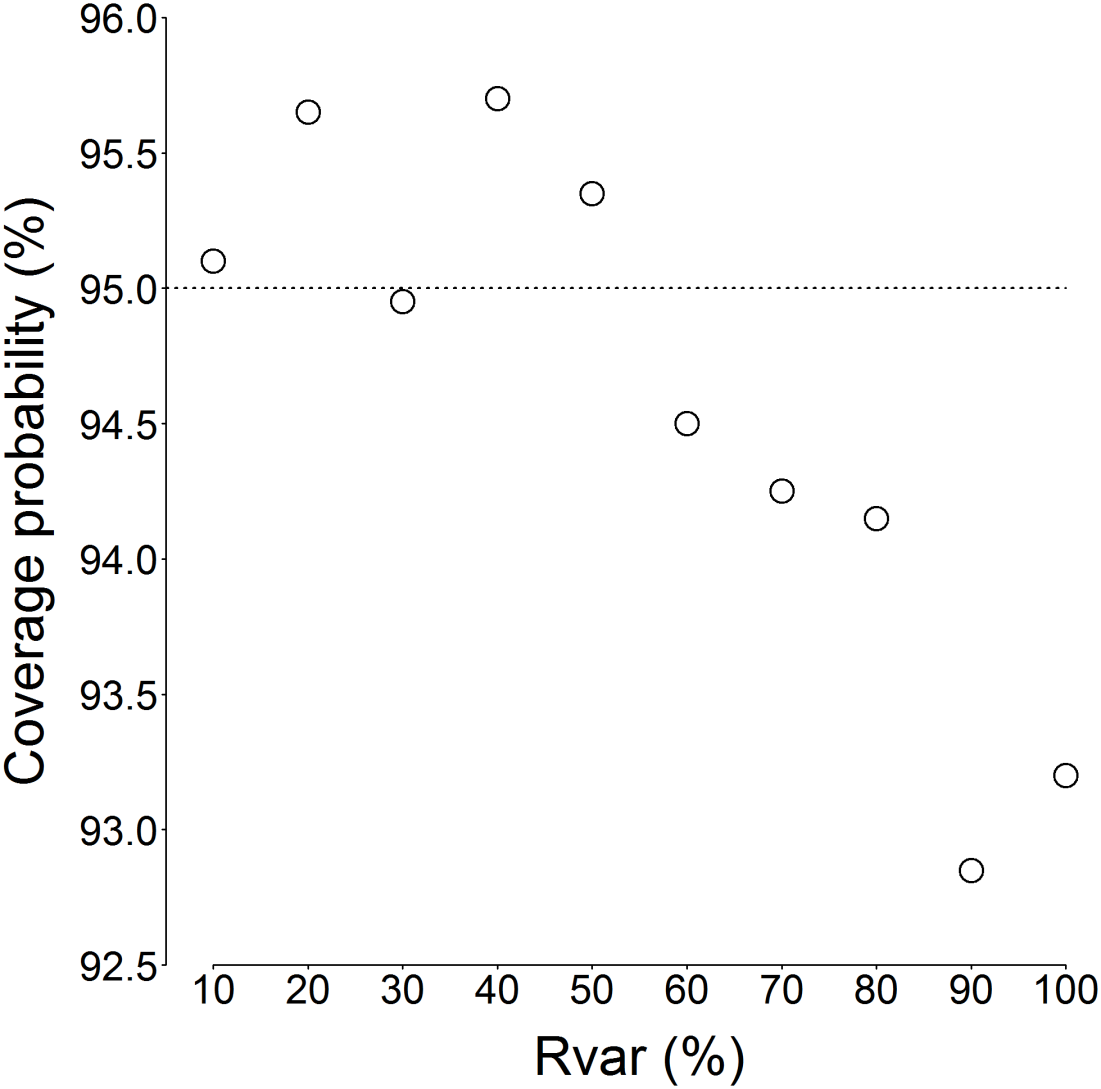
Coverage probability (%) – the percentage of simulated recruitment falling inside the 95% prediction intervals of the curvilinear regression (Eq. 7) – for each amplitude of recruitment variability (Rvar, %; see section 2.2.3).

The curvilinear regression (Eq. 7) was fitted to model outputs from each level of recruitment variability (Fig. 4). The regression successfully captured increasing recruitment variability as demonstrated by a widening of the prediction intervals (Fig. 4). The predictive performance of each regression was assessed by calculating coverage probability as the percentage of simulated recruitment values falling inside the prediction intervals (Fig. 5). The 95% prediction intervals were selected, so when a model is performing predictions inadequately, less than 95% of simulated recruitment values will fall inside the prediction intervals. Based on coverage probability, the regression performed adequately up to 50% recruitment variability (Fig. 5). Above 50% recruitment variability, the predictive performance progressively degraded with <95% of recruitment simulations falling inside the 95% prediction intervals. Under the widest range of recruitment variability (Rvar = 100%) where recruitment was randomly set between 0 and 8×10^6^ individuals, 93.2% of the absolute recruitment values fell inside the 95% prediction intervals (Fig. 5). The regression parameters obtained under Rvar = 100% are given in table 1.

**Table 1:** Parameter values for the relationship between recruitment and the minimum F40 (Eq. 7) under a simulated recruitment variability (Rvar) of 100%.

	β_0_	β_1_
Mean fit	10.0249 (2.9535×10^−3^)	1.4088 (0.2387×10^−3^)
Upper	10.9033	1.4078
Lower	9.1465	1.4098

Parameters for the 95% prediction interval are included (upper and lower). The parameter variance estimates are given in parenthesis for the mean fit.

**Figure 6:**
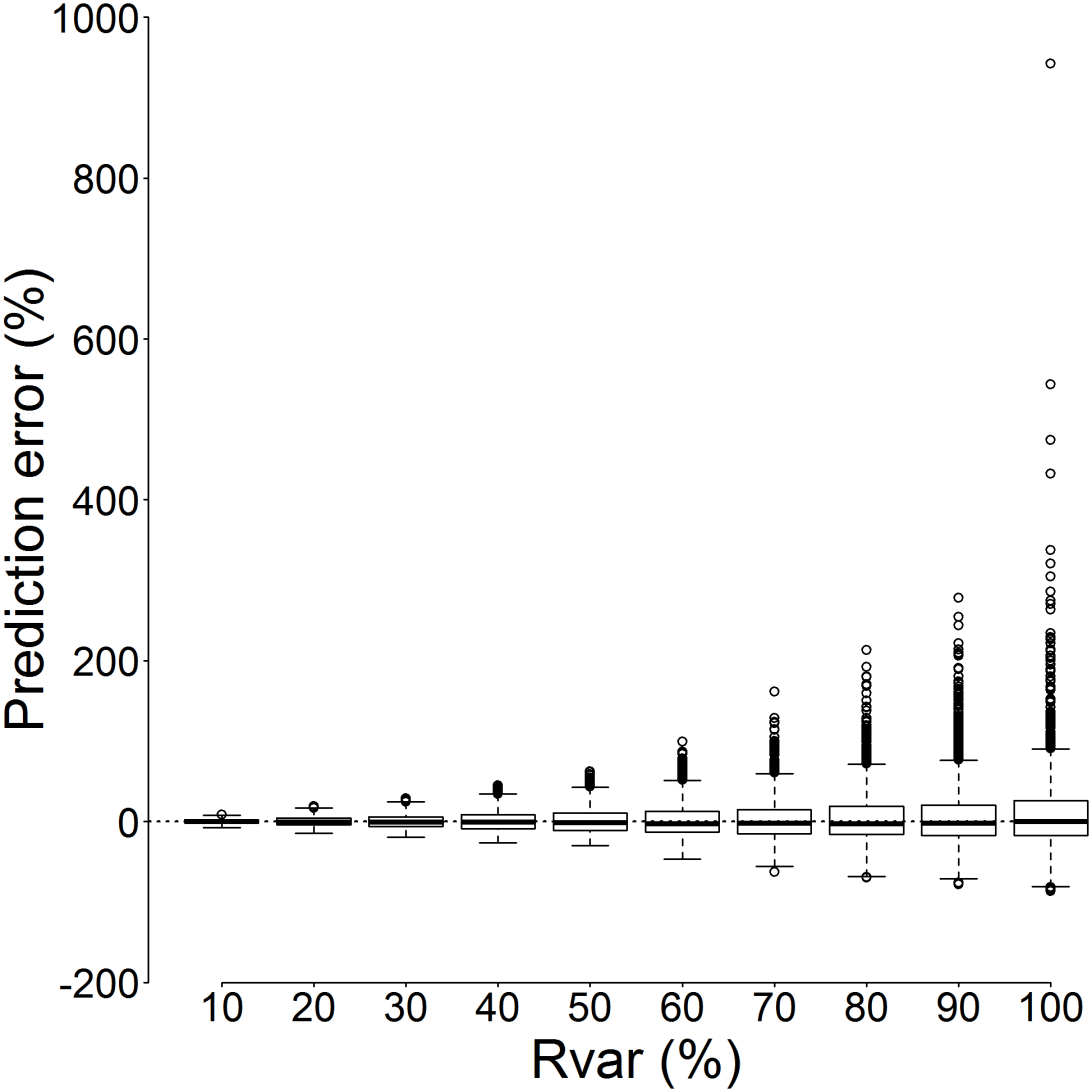
Curvilinear regression prediction error (%, Eq. 4) for each amplitude of recruitment variability (Rvar, %; see section 2.2.3). Prediction error was computed between the simulated recruitment and the recruitment predicted by the regression using the minimum F40 (Eq. 7). In this boxplot, the range of each box corresponds to the interquartile range (IQR) and the whiskers extend to an additional 1.5IQR. Values falling beyond the whiskers are marked with circles.

The range of prediction errors (Eq. 4) increased with the increasing recruitment variability from ranging between −7.4% and +8.8% at Rvar = 10% to ranging between −86.3% and +942.5% at Rvar = 100% (Fig. 6). Although the median of all prediction errors remained close to zero, the boxplots illustrate that the predictive error distribution was asymmetric, with overestimates being more prevalent. This was due to the fact that the simulated recruitment was bound between values determined by Rvar (e.g. between 0 and 8×10^6^ individuals under Rvar = 100%), while the regression could freely extrapolate estimated recruitment to higher values.

### 3.3 Multiannual Recruitment Formula

To improve predictions of recruitment, a multiannual linear regression of the log-transformed model outputs was used where explanatory variables were past values of minimum F40 (*F*40 *min*). Using three years as an example, the number of recruits released in the tenth year of simulations (*R_y_*_10_) was estimated as:
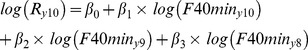
(8)


**Figure 7:**
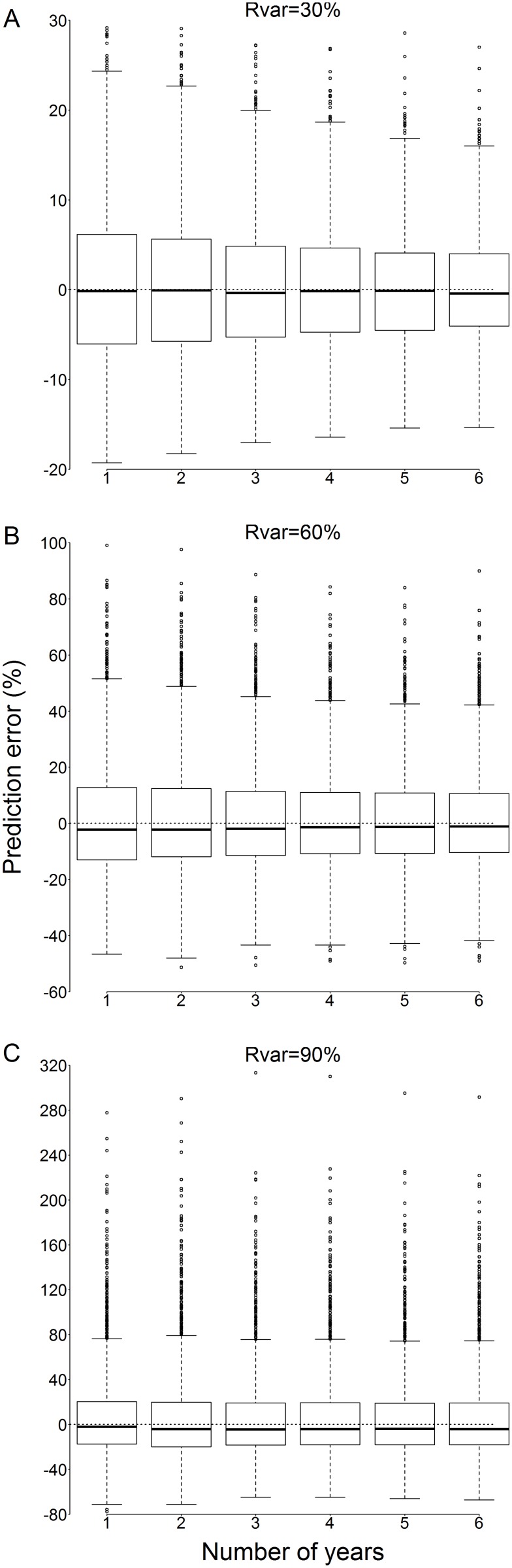
Prediction error (%, Eq. 4) computed between the simulated recruitment and the recruitment estimated with the multiannual formula using past values of minimum F40 (Eq. 8), as a function of the number of values of minimum F40 included in the regression (from 1 to 6), for three amplitudes of recruitment variability (A–C; Rvar, %; see section 2.2.3). In this boxplot, the range of each box corresponds to the interquartile range (IQR) and the whiskers extend to an additional 1.5IQR. Values falling beyond the whiskers are marked with circles.

Including past consecutive values of minimum F40 to predict recruitment narrowed the range of prediction errors for simulations under low recruitment variability (Rvar = 30%, Fig. 7A). The improvement was less evident under moderate recruitment variability (Rvar = 60%, Fig. 7B), in which case including three consecutive values of minimum F40 brought a similar improvement to when including more values. Under high recruitment variability (Rvar = 90%, Fig. 7C) the narrowing of the range of errors was almost negligible, particularly when including more than 3 consecutive values of minimum F40. The mean bias reduction (mean of absolute errors) resulting from the inclusion of three consecutive values was 16.5%, 8.3% and 3.6% under Rvar values of 30%, 60% and 90% respectively.

## Discussion

The recruitment index *F40 min* (minimum proportion of individuals smaller than 40 mm in a given year) was selected as the optimum index from six candidate indices. *F40 min* was selected as optimum because in addition to its monotonic relationship that held across a range of recruitment variability, the index-to-recruitment relationship could be expressed using simple curvilinear regression. In simulations of high recruitment variability (Rvar ≥60%, Fig. 4), the log-linear model did not perfectly capture the underlying index-to-recruitment relationship. Whilst more complex regressions may have achieved this in specific instances, it is unlikely that these models would have performed equally for all amplitudes of recruitment variability. In this research, we were seeking a model that performed well across a range of recruitment variability; in reality recruitment variability is unknown so one cannot apply a more complex model to suit high variability situations, hence a model that performed best over a range of recruitment variability was selected. Important to the process of recruitment metric selection was the underlying population model and the calculation of recruitment indices on a monthly basis, both of which will be discussed in subsequent paragraphs.

The impact of recruitment variability on length-based recruitment indices was investigated using an individual-based population model. The model captured complex population-level processes emerging from cyclical and variable recruitment by accounting for the co-existence of cohorts belonging to recruitment events of different intensities. Varying the range over which recruitment took place led to simulated pulses in krill numbers, a phenomena observed at South Georgia [3], and enabled us to test recruitment index performance against a biologically plausible, albeit simulated, krill population.

Proportional indices of recruitment such as F40 have traditionally been computed using length data available from a single survey or pooled at an annual scale [2], [7], [8], [11]–[13], [17], [20], [22], [24]. However, due to growth and mortality, length-frequencies vary at the sub-annual scale (e.g. [3], Fig. 1). Therefore, pooling length data into a single annual length-frequency distribution may conflate several underlying population processes, potentially confounding the signal produced by recruitment events. When searching for the optimum length-based recruitment index, order statistics – median and F40– were calculated on length data aggregated by month (see section 2.1). Monthly order statistics were summarised into a single annual recruitment estimate and the minimum value of F40 within a given year was found to be the optimum index of the recruitment that occurred in the summer of that year.

Increasing recruitment variability resulted in increased uncertainty in length-based recruitment indices (Figs. 3–5). Recruitment variability up to 50% was successfully captured using the 95% prediction intervals calculated from the curvilinear regression based on *F40 min* (Figs. 6, 7). True recruitment variability cannot be determined, so it is not possible a priori to select a particular regression from those determined (Fig. 4). In the absence of additional information on recruitment variability, it is recommended that the curvilinear model fitted to the widest range of recruitment variability (Rvar = 100%) is used. Under high recruitment variability, the improvement brought by the use of a multiannual formula was only minimal (Fig. 7C). Whilst under low and moderate recruitment variability, the multiannual formula yielded improved predictions, it performed poorly under high recruitment variability. Outside of the simulation, true recruitment variability is unknown so it is not possible to determine when to use such formula. Therefore the simpler single-year formula obtained under high recruitment variability is recommended to estimate annual recruitment (Table 1).

High population variability was not always accurately represented by the curvilinear regression, with less than 95% of the simulated population falling inside the 95% prediction intervals when recruitment variability exceeded 50%. Large prediction errors in situations of high recruitment variability suggest length-based indices are of limited value, a fact that has been previously raised in the case of fish stock assessments (e.g. [31]). More positively, the approach presented here provides an objective mechanism through which to assess the utility of recruitment indices, and which enable researchers to incorporate uncertainty when considering the links between recruitment and environmental drivers. Furthermore, our results indicated that using these indices to track recruitment events could provide an objective approach to estimate the magnitude and confidence associated with these events. In particular, the uncertainty around recruitment estimates appeared to increase with the magnitude of the recruitment event, suggesting that whilst being beneficial, correlation analysis between estimated recruitment and environmental drivers will be more difficult for highly uncertain, large recruitment events.

Since a recruitment event will impact the population size structure over several years, a length-frequency distribution at a given instant may carry information on recruitment events that occurred in previous years. Including information on the population size structure over the years preceding a given recruitment event could improve the accuracy of that recruitment estimate. Improvement in length-based recruitment estimates via multiannual estimates has been suggested in previous studies (e.g. [11]), and was successfully demonstrated here when recruitment variability was less than 60% (Fig. 7). In this study, improvement in the prediction of recruitment was itself dependent on recruitment variability since increased recruitment variability weakened the link between current and previous recruitment indices. Nevertheless, an improvement in the accuracy of the recruitment predictions was obtained under all ranges of recruitment variability, and, given that the actual variability of recruitment in the real world is unknown adopting such an approach could be beneficial. However, as stated above, the improvement was only minimal (mean bias reduction of 3.6% under Rvar = 90%) under high recruitment variability.

In addition to analysis of ecological significance, the results presented here could be beneficial to the management of the krill fishery. Stock assessment models are designed to estimate population parameters by determining the set of parameters enabling the best fit between simulations and observations, including length-frequencies distributions (e.g. [19]). Stock assessment models could benefit from the method of recruitment estimation presented here for their initialisation through a time-series of estimated recruitment. Additionally, model verification could be performed through a comparison of stock-assessment and simulation model outputs (Eq. 7).

The underlying model used to simulate population dynamics was parameterised using values drawn from the published literature. In order to establish the baseline response, the model structure was intentionally kept simple and made to replicate behaviour of an average population sampled homogeneously. More complex modelling schemes could be devised in the future to account, in particular, for biological variability, such as inter-annual changes in growth, mortality, recruitment timing and duration, as well as spatial and temporal biases in sampling effort and investigate their impact on length-based recruitment estimates. In addition, recruitment was set to occur each year in simulations independently of the status of the adult population. A complete mechanistic life cycle model could be formulated in the future to account for the maturation of individuals in the population and their participation in the spawning stock. Such level of detail would enable investigating processes affecting recruitment variability such as generation time, lifespan and age at maturity. The approach of decoupling recruitment from the reproductive status of the population is robust in that it makes no assumptions about the links between the two and enables the performance of recruitment indices to be assessed without formulating hypothesis on these links.

Despite the relatively simple model structure, the findings presented still bring a significant improvement in our ability to extract information from length measurements. The modelling approach described here, could be applied to any species targeted by a length-based survey, provided a temporal coverage enabling the determination of the bounds of the chosen length-based index (e.g. the determination of the minimum F40 in a given year in our case). A potential future application of this approach is the estimation of recruitment based on time-series of krill length measurements collected as part of the CCAMLR Scheme of International Scientific Observation, which could unveil crucial information on the population dynamics of *Euphausia superba*.

## Supporting Information

Figure S1 NOAA Optimum Interpolation of monthly Sea Surface Temperature V2 (within 65°S to 53°S and 64°W to 34°W), showing the mean (line), range (grey area), and the sinusoidal fitted function (–1≤ *SV_(t)_* ≤ +1; dotted line) used in the seasonally varying von Bertalanffy (vB) growth sub-model.
(TIF)

Figure S2 *Euphausia superba*, model outputs. Simulated length-at-age (A), showing the mean (solid line), standard deviation (grey area) and extremes (dotted lines); selected levels of availability for capture (Eq. 2, see main text) are shown as horizontal lines and the post-recruitment durations in months for the mean length to reach levels of 5% and 50% are indicated. The remainder of panels (B–D) show model outputs for the last 25 months of simulations overlayed on the recruitment frequency distribution (grey histograms). The number of individuals (B) in the population (solid line, left y-axis) and in captures (dotted line, right y-axis) are shown; the captured individuals being those included in the computation of the monthly median length (mm; C) and proportion of individuals smaller than 40 mm (F40, %; D). In the last year of simulation, the maximum (upward triangle) and minimum (downward triangle) of the median length and F40 are shown, as well as the span of values (vertical double arrow).
(TIF)

Information S1 Krill growth model equations and outputs.
(DOCX)
