# Supplementary figures and images for: Investigating the Effect of Recruitment Variability on Length-Based Recruitment Indices for Antarctic Krill Using an Individual-Based Population Dynamics Model
Source: PLoS One. 2014 Dec 3;9(12):e114378. doi: 10.1371/journal.pone.0114378 (PMC4254992; doi:10.1371/journal.pone.0114378)

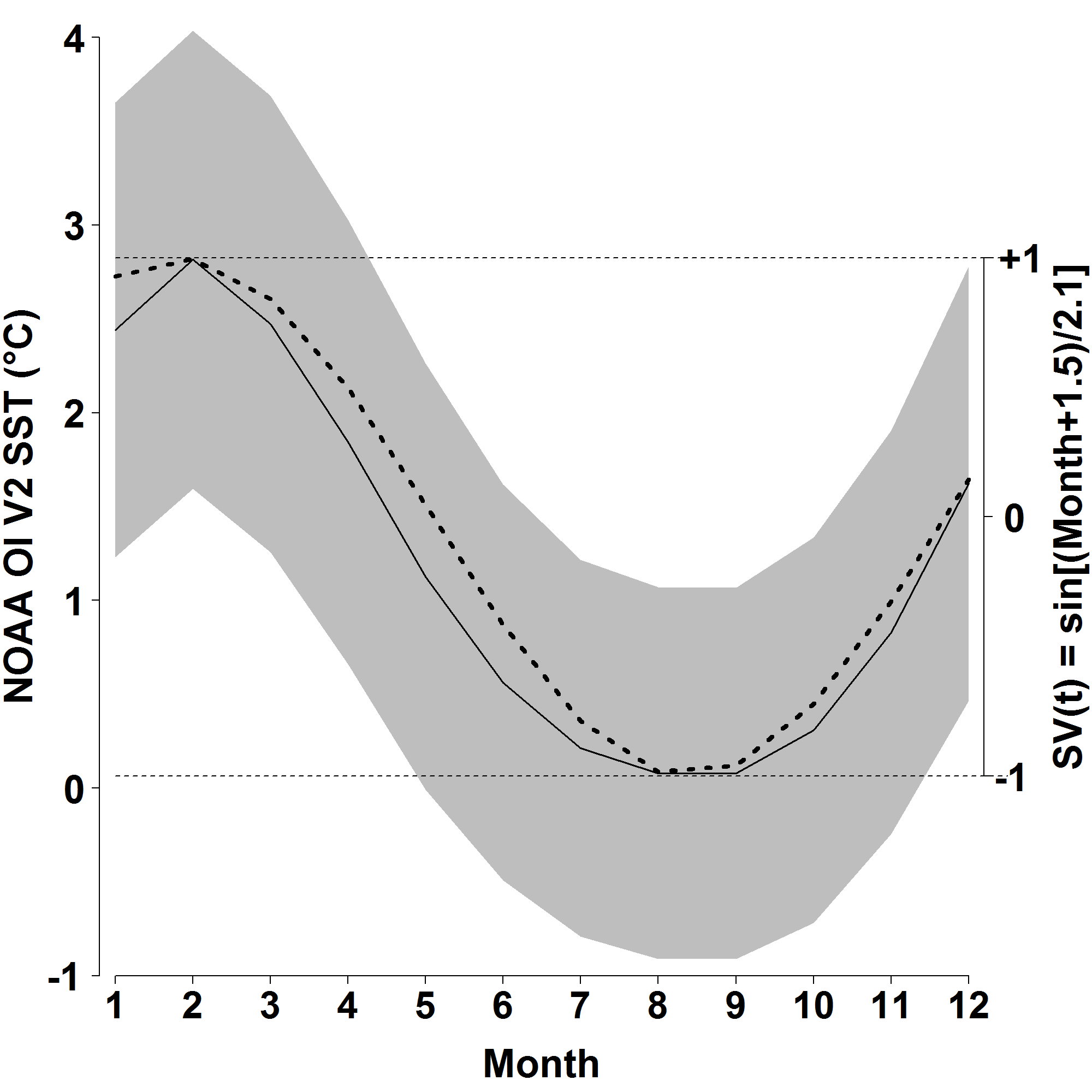

Supplement: Figure S1 — NOAA Optimum Interpolation of monthly Sea Surface Temperature V2 (within 65°S to 53°S and 64°W to 34°W), showing the mean (line), range (grey area), and the sinusoidal fitted function (–1≤ SV(t) ≤ +1; dotted line) used in the seasonally varying von Bertalanffy (vB) growth sub-model. (TIF) [file pone.0114378.s001.tif]

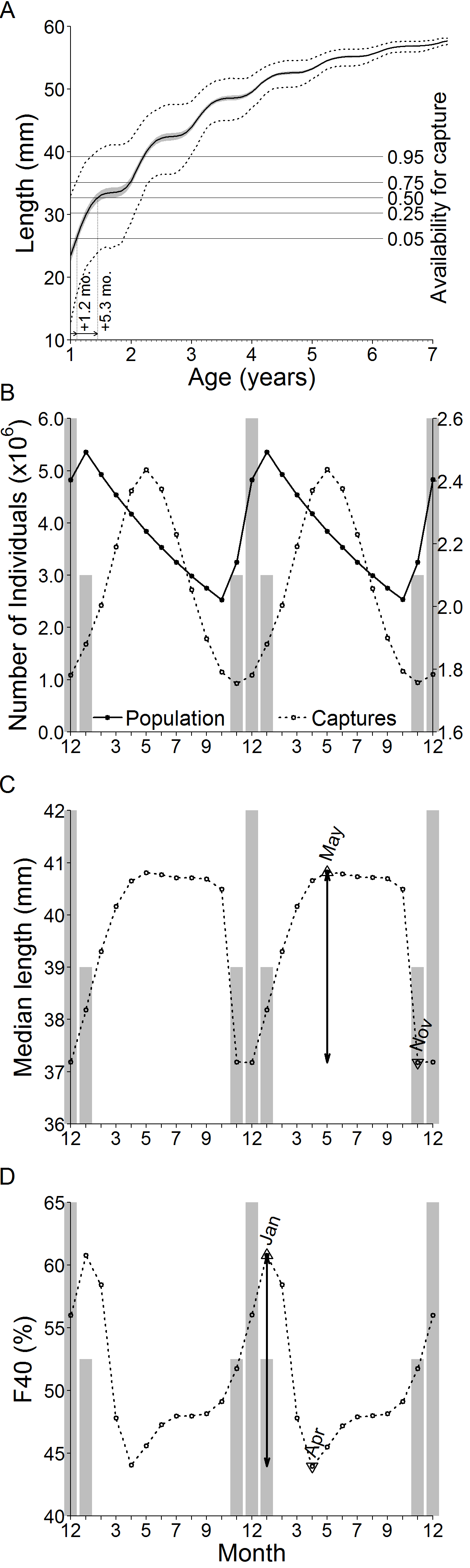

Supplement: Figure S2 — Euphausia superba, model outputs. Simulated length-at-age (A), showing the mean (solid line), standard deviation (grey area) and extremes (dotted lines); selected levels of availability for capture (Eq. 2, see main text) are shown as horizontal lines and the post-recruitment durations in months for the mean length to reach levels of 5% and 50% are indicated. The remainder of panels (B–D) show model outputs for the last 25 months of simulations overlayed on the recruitment frequency distribution (grey histograms). The number of individuals (B) in the population (solid line, left y-axis) and in captures (dotted line, right y-axis) are shown; the captured individuals being those included in the computation of the monthly median length (mm; C) and proportion of individuals smaller than 40 mm (F40, %; D). In the last year of simulation, the maximum (upward triangle) and minimum (downward triangle) of the median length and F40 are shown, as well as the span of values (vertical double arrow). (TIF) [file pone.0114378.s002.tif]
