# Supplementary material for: Investigating the Effect of Recruitment Variability on Length-Based Recruitment Indices for Antarctic Krill Using an Individual-Based Population Dynamics Model
Source: PLoS One. 2014 Dec 3;9(12):e114378. doi: 10.1371/journal.pone.0114378 (PMC4254992; doi:10.1371/journal.pone.0114378)
Supplement: Information S1 — Krill growth model equations and outputs. (DOCX) [file pone.0114378.s003.docx]

Supporting Information S1

Krill growth model

Various approaches to modelling krill growth exist, ranging from simple von Bertalanffy (vB) formulations (e.g. [1–3]) to detailed bioenergetics models (e.g. [4–6]). We adopted a similar approach to Siegel (1987 [7]) whereby a sinusoidal term is included in a vB formulation to account for temperature-dependent seasonality (i.e. null growth in winter, fast growth in summer). The model was used to update the length of each individual krill at each (monthly) time-step in simulations. The vB growth model was applied using a transformation of the vB equation into an instantaneous growth rate (e.g. [8]):

$L_{(t)}=L_{(t-1)}+\frac{k(L_{\infty}-L_{(t-1)})}{12}$ (s1)

where *L_(t)_* is the length (mm) of a given individual at time *t*, and with a denominator of 12 to convert the original annual formulation to the monthly resolution of our model (values of *k*=0.45 and *L_∞_*=60mm were taken from Rosenberg et al 1986 [1]).

Satellite-derived sea surface temperature NOAA OI SST V2 data provided by the NOAA/OAR/ESRL PSD (available at http://www.esrl.noaa.gov/psd/ [9]) at a monthly resolution, within an area covering the distribution of krill fishing in the CCAMLR Convention Area (53°S to 65°S ; 34°W to 64°W ), from Dec. 1981 to Dec. 2013 were fitted with a sinusoidal function (Fig. S1), defined by the month of the year, and used as a seasonally varying factor (*SV*) as follows:

$L_{(t)}=L_{(t-1)}+\frac{k(L_{\infty}-L_{(t-1)})}{12}\times({SV}_{(t)}+1)$ (s2)

with:

${SV}_{(t)}=sin(\frac{{month}_{(t)}+1.5}{2.1})$ (s3)

where *month_(t)_* is the month number (from 1 to 12) at time *t*. Since *SV* varies between –1 and +1, the seasonally varying vB formulation (Eq. s2) was scaled to give zero growth at the coldest time of year and growth multiplied by a factor of 2 at the warmest part of the cycle. Krill population dynamics were simulated over 10 years, with a constant annual recruitment of 4×10^6^ individuals (2×10^6^ recruits in December, and 1×10^6^ in November and January).

*Length-at-age*

The simulated length-at-age tended to an asymptote at *ca.* 60mm after 7 years (Fig. S2A). Since individuals are recruited in early summer their length-at-age initially increased rapidly before reaching a first plateau during the subsequent winter. The mean length-at-age took 1.2 months to reach a 5% availability for capture (i.e. probability for individuals to be captured by the fishery), and 5.3 months to reach a 50% availability.

*Number of individuals*

The number of individuals in the simulated population fluctuated between *ca.* 2.5 and 5.5 million, with rapid increases due to recruitment and slower decreases due to mortality (Fig. S2B). The number of individuals captured peaked at *ca*. 2.4 million and the timing of the maximum capture was 4 months later than the maximum of population count.

*Median length and F40*

Due to the sinusoidal pattern in the number of individuals captured the monthly median length and proportion of individuals smaller than 40mm (F40, %) both displayed cyclical trends (Fig. S2C, D). The median length moved rapidly to a minimum between November and December coincident with the timing of recruitment, whereas the F40 reached a maximum in January followed by a minimum in April.

**Supporting information S1 references**

1. Rosenberg AA, Beddington JR, Basson M (1986) Growth and longevity of krill during the first decade of pelagic whaling. Nature 324: 152–154.

2. Murphy E, Reid K (2001) Modelling Southern Ocean krill population dynamics: biological processes generating fluctuations in the South Georgia ecosystem. Mar Ecol Prog Ser 217: 175–189. doi:10.3354/meps217175.

3. Kawaguchi S, Finley L, Jarman S, Candy S, Ross R, et al. (2007) Male krill grow fast and die young. Mar Ecol Prog Ser 345: 199–210. doi:10.3354/meps06934.

4. Hofmann EE, Lascara CM (2000) Modelling the growth dynamics of Antarctic krill Euphausia superba. Mar Ecol Prog Ser 194: 219–231.

5. Fach B, Meyer B, Wolf-Gladrow D, Bathmann U (2008) Biochemically based modeling study of Antarctic krill Euphausia superba growth and development. Mar Ecol Prog Ser 360: 147–161. doi:10.3354/meps07366.

6. Lowe AT, Ross RM, Quetin LB, Vernet M, Fritsen CH (2012) Simulating larval Antarctic krill growth and condition factor during fall and winter in response to environmental variability. Mar Ecol Prog Ser 452: 27–43. doi:10.3354/meps09409.

7. Siegel V (1987) Age and growth of Antarctic Euphausiacea (Crustacea) under natural conditions. Mar Biol 96: 483–495.

8. Candy S, Kawaguchi S (2006) Modelling growth of Antarctic krill. II. Novel approach to describing the growth trajectory. Mar Ecol Prog Ser 306: 17–30.

9. Reynolds RW, Rayner NA, Smith TM, Stokes DC, Wang W (2002) An Improved In Situ and Satellite SST Analysis for Climate. J Clim 15: 1609–1625.
